# Breastfeeding and IQ Growth from Toddlerhood through Adolescence

**DOI:** 10.1371/journal.pone.0138676

**Published:** 2015-09-25

**Authors:** Sophie von Stumm, Robert Plomin

**Affiliations:** 1 Department of Psychology, Goldsmiths University of London, London, United Kingdom; 2 King's College London, MRC Social, Genetic & Developmental Psychiatry Centre, Institute of Psychiatry, Psychology & Neuroscience, London, United Kingdom; Universitat Wien, AUSTRIA

## Abstract

**Objectives:**

The benefits of breastfeeding for cognitive development continue to be hotly debated but are yet to be supported by conclusive empirical evidence.

**Methods:**

We used here a latent growth curve modeling approach to test the association of breastfeeding with IQ growth trajectories, which allows differentiating the variance in the IQ starting point in early life from variance in IQ gains that occur later in childhood through adolescence. Breastfeeding (yes/ no) was modeled as a direct predictor of three IQ latent growth factors (i.e. intercept, slope and quadratic term) and adjusted for the covariates socioeconomic status, mother's age at birth and gestational stage. Data came from the Twins Early Development Study (TEDS), a prospective cohort study of twins born between 1996 and 1994 in the United Kingdom, who were assessed 9 times on IQ between age 2 and 16 years (N = 11,582).

**Results:**

Having been breastfed was associated with a small yet significant advantage in IQ at age 2 in girls (β = .07, CI 95% from 0.64 to 3.01; N = 3,035) but not in boys (β = .04, CI 95% from -0.14 to 2.41). Having been breastfeeding was neither associated with the other IQ growth factors in girls (slope: β = .02, CI 95% from -0.25 to 0.43; quadratic: β = .01, CI 95% from -0.02 to 0.02) nor in boys (slope: β = .02, CI 95% from -0.30 to 0.47; quadratic: β = -.01, CI 95% from -0.01 to 0.01).

**Conclusions:**

Breastfeeding has little benefit for early life intelligence and cognitive growth from toddlerhood through adolescence.

## Introduction

Many empirical studies have investigated if being breastfed in early life benefits later cognitive development [1]. An association between breastfeeding and cognition is plausible because long-chain polysaturated fatty acids (PUFA), which are present in human breast milk but not in milk from animals or formula, enhance neurodevelopment [2]. However, the evidence on the association between breastfeeding and cognitive growth is to date inconsistent and inconclusive, with some studies reporting a positive relationship and others failing to detect such effects after adjusting for relevant covariates. A recent comprehensive review of 84 relevant studies on breastfeeding practices and intelligence concluded that any observed associations between the two were best explained by residual confounding [1]. Thus, the relationship between breastfeeding and intelligence may not be causal in nature but instead reflected the interconnection of favorable variables associated with breastfeeding, such as advantaged family socioeconomic status (SES) and higher parental intelligence [3].

To disentangle ‘true’ and ‘confounding’ effects in the breastfeeding-intelligence association, it is important to differentiate children’s differences in early life intelligence–that is, at the beginning of their cognitive growth trajectories–from the differences that children show in cognitive growth or intelligence gains over time [4]. If there were nutritional benefits of breastfeeding for cognitive growth, breastfeeding should be more strongly associated with early life cognitive ability or IQ starting points but to a lesser degree with children’s later intelligence gains [4]. However, if breastfeeding was mainly related to long-term cognitive development rather than initial intelligence, the association was likely to result from confounding and be attributable to breastfeeding’s interrelatedness with other variables that exert favorable influences on development [4]. Huang and colleagues tested this hypothesis recently in data from the Child Development Supplement of the Panel Study of Income Dynamics (CDS), a panel sample of 2,784 children, who were initially aged 0 to 12 years and assessed up to three times on cognitive ability over a study period of 10 years. Breastfeeding was modeled as a dichotomous predictor of children’s cognitive ability test scores at each assessment wave, adjusting for relevant covariates. The results showed that children who had been breastfed had significantly higher initial intelligence test scores than those who had not been breastfed. However beyond that, breastfeeding was not associated with cognitive growth trajectories. That is, the long-term cognitive development of breastfed and not breastfed children was similar, supporting the nutritional benefits hypothesis, even though the initial breastfeeding gap persisted over time. Huang and colleagues concluded that breastfeeding had a ‘true’ effect on cognitive development that was not eliminated by later life experiences and thus, was not to be attributed to residual confounding.

In the current study, we seek to replicate and extend Huang and colleagues’ earlier findings, overcoming four limitations of their original investigation. First, the CDS study sample included children with a wide age range (0 to 12 years); thus, early IQ development could only be studied for part of the sample. By comparison, we analyze data from the Twins Early Development Study (TEDS), a prospective cohort twin sample that is demographically representative of the United Kingdom and whose members were born between 1994 and 1996 [5]. Second, the intelligence of the CDS children was assessed at most three times and at different ages during the 10-year study period of 10 years, but for some children only one or two valid cognitive scores were available [4]. By comparison, TEDS children were tested 9 times on intelligence between the ages of 2 and 16 year; thus, they were assessed at the same ages and at least three times during the 14-year study duration. Also, TEDS includes a much larger sample of children than CDS (11,582 vs. 2,784). Our large, longitudinally assessed sample allows testing latent growth curve (LGC) models, which differentiate variance in latent growth factors that represent children’s differences in early life intelligence and systematic changes or gains in intelligence over time [6]. Third, data on breastfeeding practices in CDS were collected from mothers at the study’s first assessment wave, that is, 0 to 12 years after the actual event. Mothers’ recall of breastfeeding has been shown to be most reliable and valid within the first three years the event [7]. In TEDS, breastfeeding data also rely on mothers’ recall at the first assessment wave, but this took place within two years of the twins’ birth, suggesting that the breastfeeding data used in the current study will be less affected by measurement error. Fourth, twins’ cognitive development is similar to that of singletons [8], and twin studies make it possible to confirm results by testing two directly comparable samples of siblings, each consisting of one twin randomly chosen from a pair. This possibility of direct replication is not provided by cohort studies of unrelated children. Finally, we also explore here gender differences in our LGC analysis of cognitive development in TEDS, which had not been previously addressed.

## Methods

### Sample

The Twins Early Development Study (TEDS) recruited initially over 15,000 families of twins born in England and Wales between 1994 and 1996. While some have since been lost due to attrition, the sample has remained representative of the U.K. population [5]. For the current study, we excluded all twins from the analysis who suffered from severe medical problems during pregnancy, currently or at birth (e.g. postnatal surgery; N = 1,670); whose first language was not English (N = 520); and who were assessed on intelligence fewer than three times between the ages of 2 and 16 years (N = 15,786). With that, the analysis sample included 11,582 children (6,059 girls) with 4115 monozygotic and 7450 dizygotic twin pairs. In comparisons between the analysis sample and those children excluded from our analyses, we found significant differences in mean SES but not in SES variance, which is of primary interest in the current study and statistical models. Furthermore, 60% of mothers in the analysis sample and 62% in the sample of children excluded from the analyses reported to have breastfed, suggesting no attrition effect for this key variable.

### Measures

#### Intelligence (IQ)

The twins were assessed at 2, 3, 4, 7, 9, 10, 12, 14 and 16 years on intelligence, using parent-administered tests and ratings of ability at ages 2, 3, and 4, and a mixture of web-based, telephone-based, and parent-administered tests at later ages. At each testing age, twins completed at least two state-of-the-art ability tests. The tests have been described in detail elsewhere [9] and are only briefly reviewed here.

#### Measures at ages 2, 3, and 4

Parent-administered tests and parent-reported observations (PARCA) were used to assess verbal and nonverbal cognitive abilities. These measures have been previously validated against standard infant cognitive ability tests administered by trained testers [10,11]: the age 2 version of PARCA correlated with the Mental Scale of the Bayley Scales of Infant Development II (BSID-II) between .43 and .51 in 106 two-year old children [10], and the age 3 version of PARCA correlated with the General Cognitive Index of the McCarthy Scales of Children’s Abilities .46 in 85 three-year old children [11]. In TEDS, nonverbal cognitive performance was assessed using age-appropriate PARCA versions, while verbal ability measures included vocabulary and grammar as assessed by parent reports for the CDI-III, an extension of the short form of the MacArthur Communicative Development Inventories: Words and Sentences [12].

#### Measures at age 7

Children were tested on verbal and nonverbal abilities by telephone [13]. Prior to the telephone call, parents were sent a booklet of test items along with testing instructions. The booklet contained two verbal tests: the Similarities subtest and the Vocabulary subtest from the Wechsler Intelligence Scale for Children (WISC-III-UK) [14]; and two nonverbal tests: the Picture Completion subtest from the WISC-III-UK and Conceptual Grouping from the McCarthy Scales of Children's Abilities [15].

#### Measures at age 9

Participants were mailed a test booklet with two verbal and two nonverbal tests to be administered under the supervision of the parent, who had received a corresponding instruction booklet. The verbal tests comprised vocabulary and general knowledge tests adapted from the multiple-choice version of the WISC-III-UK [14]. The nonverbal tests included a Puzzle test adapted from the Figure Classification subtest of the Cognitive Abilities Test 3 (CAT3) [16] and a Shapes test also adapted from the CAT3 Figure Analogies subtest [17].

#### Measures at age 10

Testing was web-based, and children completed two verbal and two non-verbal tests using their home computers [18]. Tests were drawn from the WISC-III-PI, including Multiple Choice Information (General Knowledge), Vocabulary Multiple Choice, and Picture Completion [14], and from Raven's Standard Progressive Matrices [19].

#### Measures at age 12

Testing was web-based and conducted using home computers with age-matched versions of the two verbal and two non-verbal tests previously used at age 10 [14, 19, 20].

#### Measures at age 14

Twins completed two web-based tests at their home computers: WISC-III-PI Vocabulary Multiple Choice for 14-year olds [10] and Raven's Progressive Matrices [19].

#### Measures at age 16

Twins completed web-based adaptations of Raven’s Standard and Advanced Progressive and the Mill-Hill Vocabulary Scale using their home computers [19, 21].

#### Breastfeeding

At first contact when the twins were on average 18 months old, mothers were asked a whether they had breastfed their twins (yes/ no). The question was asked including both twins and not for each twin separately. Mothers also reported how long they had breastfed each twin in days. The correlation of breastfeeding duration across twins was .97.

#### Socioeconomic status (SES)

Parental education and occupation (mother’s and father’s highest educational qualification and job status) were recorded at the first contact with the families, when twins were 18 months old, and again when they were 7 years old. Family income was assessed when the twins were 9 years old. A composite of parental education and occupation at twins’ age of 18 months correlated at .77 with a composite of parental education and occupation at twins’ age 7, which in turn correlated at .57 with family income at twins’ age 9, suggesting that SES was relatively stable over time in TEDS [9]. Data from the assessment at 18 months were used in cases where information at age 7 was missing; for all others, records of parental education and occupation at age 7 were employed. Summary SES composites were created as a unit-weighted sum of the education, occupation, and income after mapping to a standard normal distribution with the rank-based van der Waerden transformation [9].

#### Maternal age

At the first contact, mothers reported their age in years when they gave birth to the twins.

#### Gestational age

At the first contact, mothers reported twins’ gestational age as an estimate based on their last menstrual period before giving birth.

### Analysis

First, the first principal component was extracted from the IQ tests scores at each assessment wave and then transformed into IQ scores (mean = 100, SD = 15). The comparability of IQ scores over time is here theoretically inferred, because intelligence was assessed at each measurement occasion with multiple, well validated tests that are thought to capture identical constructs, yielding invariant common variance factors of intelligence, even if different tests were administered using different methods at different times or ages. Furthermore, principal component scores are standardized (i.e. z-scores), ensuring that scores’ variances are invariant across time or assessment occasions. Previously, multiple first factors extracted from cognitive test batteries were shown to be invariant in adults [22, 23], although the invariance of such factors in children or over the course of time has not been established.

Second, LGC models were fitted to differentiate variance in intelligence that is stable over time from changes across assessment occasions [6]. Thus, LGC factors are extracted that describe a sample’s average IQ starting point, typically referred to as intercept, and systematic IQ changes, known as slope and quadratic term. To determine the correct number of LGC factors, the fit (i.e. χ²(df)) of LGC models with one (intercept), two (slope) and three (quadratic term) growth factors were compared. The intercept represented the mean level of the twins’ IQ at the first assessment (i.e., at age 2 years); the slope referred to the average rate of linear change in IQ over time; and the quadratic term captured the non-linear acceleration or deceleration of the growth trend (i.e. systematic curvilinear change not accounted for by the slope). At each age, the IQ score loadings on the intercept were fixed to 1, and those on the slope were defined as 0, 1, 2, 5, 7, 8, 10, 12, and 14, representing *time periods* in years between each assessment point, ranging in *real time* from 2 to 16 years. With that, the intercept was defined where the slope had a zero loading (i.e. at age 2). Loadings on the quadratic term were the square of the slope loadings.

In a third step, the baseline LGC model was extended to investigate if boys and girls differed in their cognitive development. Here, an initial model only equated the LGC factor structure across two groups of boys and girls, in line with the LGC model specifications, and the fit of this ‘unrestricted’ model was then compared to the fit of a more ‘restricted’ model that also held intercepts, means and residuals equal across groups. A significant difference between the ‘unrestricted’ and the ‘restricted’ model fits indicates that boys and girls differ systematically in their LGC factors and thus that samples should be analyzed separately for each gender.

In the fourth step of the analysis, we added to our models, in order, SES, mothers’ age at birth, gestational age, and breastfeeding as time-invariant covariates of the LGC factors. Thus, we specified each of these variables to have direct effects on the LGC factors, with the order of entry ensuring that any associations observed for breastfeeding were not attributable to the confounders. In line with Huang et al.’s previous findings, we predicted an association of breastfeeding with the intercept but not with the slope or quadratic term. In other words, breastfeeding was expected to be associated with differences in the IQ starting point but not with long-term IQ changes.

All models were fitted to an exploration sample of one twin randomly chosen from a pair (*N* = 5,800; 360 girls and 2740 boys) and subsequently to a replication sample of the co-twins (*N* = 5,782; 3053 girls and 2729 boys). In addition to enabling direct replication of our findings across two samples, randomly selecting one twin from a pair per sample also ensured that model fit statistics were not erroneously inflated because of the dependence of observations (i.e. relatedness of twins). We used full information maximum likelihood estimation (FIML) assuming data to be missing at random [24]. Several fit indices evaluated the models’ goodness of fit, including the model χ² test, the Comparative Fit Index (CFI), the Tucker–Lewis Index (TLI), and the RMSEA.

## Results

### Descriptive Statistics

Across samples, 3574/ 3572 children were reported to have been breastfed, resulting in about 62% of children in the analysis sample having been breastfed in early life, with an average duration of almost 4 months. Mothers’ age at birth ranged from 15.70 years to 50.69 years with a mean of 31.15 (SD = 4.69). Twins’ gestational age ranged from 27 to 43 weeks, with a mean of 36.52 (SD = 2.38). Data for all continuous variables, including IQ and covariates, were normally distributed. Table 1 shows the correlations of IQ scores from age 2 to 16 years; corresponding IQ means and SD did not differ across twin subsamples (*p* > .05 in all cases). IQ scores were positively inter-correlated with stronger associations between more proximate assessments, in line with other studies [25] Note that that correlations at age 4, 7 and 9 years are relatively low in the current sample, reflecting changes in the IQ assessment methods (i.e. parent- versus phone- and web-administered) but also real changes in IQ that are likely to occur at these ages because of other environmental influences (e.g. staring primary and secondary school).

**Table 1:** Correlations between IQ scores from age 2 to 16 years in two subsamples of TEDS.

	N_Twin1_	IQ 2	IQ 3	IQ 4	IQ 7	IQ 9	IQ 10	IQ 12	IQ 14	IQ16	N_Twin2_
**IQ 2**	4211	-	. 67	. 57	. 26	. 23	. 19	. 18	. 18	. 18	4235
**IQ 3**	4142	. 67	-	. 70	. 34	. 34	. 29	. 25	. 28	. 22	4157
**IQ 4**	4786	. 56	. 69	-	. 33	. 34	. 27	. 28	. 22	. 23	4812
**IQ 7**	4289	. 23	. 31	. 31	-	. 43	. 41	. 45	. 43	. 40	4306
**IQ 9**	3015	. 25	. 35	. 33	. 41	-	. 56	. 54	. 47	. 42	3001
**IQ 10**	2439	. 23	. 31	. 27	. 41	. 57	-	. 61	. 50	. 45	2449
**IQ 12**	3236	. 18	. 27	. 29	. 45	. 56	. 63	-	. 63	. 57	3208
**IQ 14**	2431	. 21	. 26	. 23	. 40	. 46	. 51	. 62	-	. 62	2456
**IQ 16**	2181	. 21	. 26	. 22	. 42	. 45	. 50	. 59	. 64	-	2183

*Note*. Correlations below the diagonal are based on children from subsample 1; correlations above the diagonal refer to children from subsample 2.

### Baseline Latent Growth Curve Models

To determine the number of latent growth factors that best represented the variance in intelligence from age 2 to 16 years, we fitted one-, two- and three-factor LGC models. The intercept-only model (twin 1: χ²(43) = 3514.50; CFI = .675; TLI = .728; RMSEA = .118 with CI of 90% .115 to .121; twin 2: χ²(43) = 3445.10; CFI = .680; TLI = .733; RMSEA = .117 with CI of 90% .114 to .120) had a worse fit than the two-factor LGC model (twin 1: χ²(40) = 1109.33; CFI = .900; TLI = .910; RMSEA = .068 with CI of 90% .064 to .071; twin 2: χ²(40) = 1115.52; CFI = .896; TLI = .906; RMSEA = .069 with CI of 90% .066 to .073) with χ²_*difference*_ (3) = 2405.17 and 2329.58, respectively (*p* < .001 in both cases). The two-factor model in turn had a worse fit than a three-factor LGC model (twin 1: χ² (36) = 578.15; CFI = .949; TLI = .949; RMSEA = .051 with CI of 90% .047 to .055; twin 2: χ² (36) = 612.15; CFI = .946; TLI = .946; RMSEA = .053 with CI of 90% .049 to .056) with χ²_*difference*_ (4) = 531.18 and 503.37, respectively (*p* < .001 in both cases). A multi-group model that restricted all path parameters to be equal across the two TEDS subsamples confirmed that model outcomes were invariant across twin samples. Thus, variances in IQ from age 2 to 16 years were best explained by an intercept (average starting point), and factors of linear (slope) and non-linear (quadratic term) change. The mean of the intercept was close to 100, while the means of the slope and quadratic term were close to 0, reflecting the norming of the IQ data at each assessment wave with a mean of 100 and an SD of 15.

**Fig 1:**
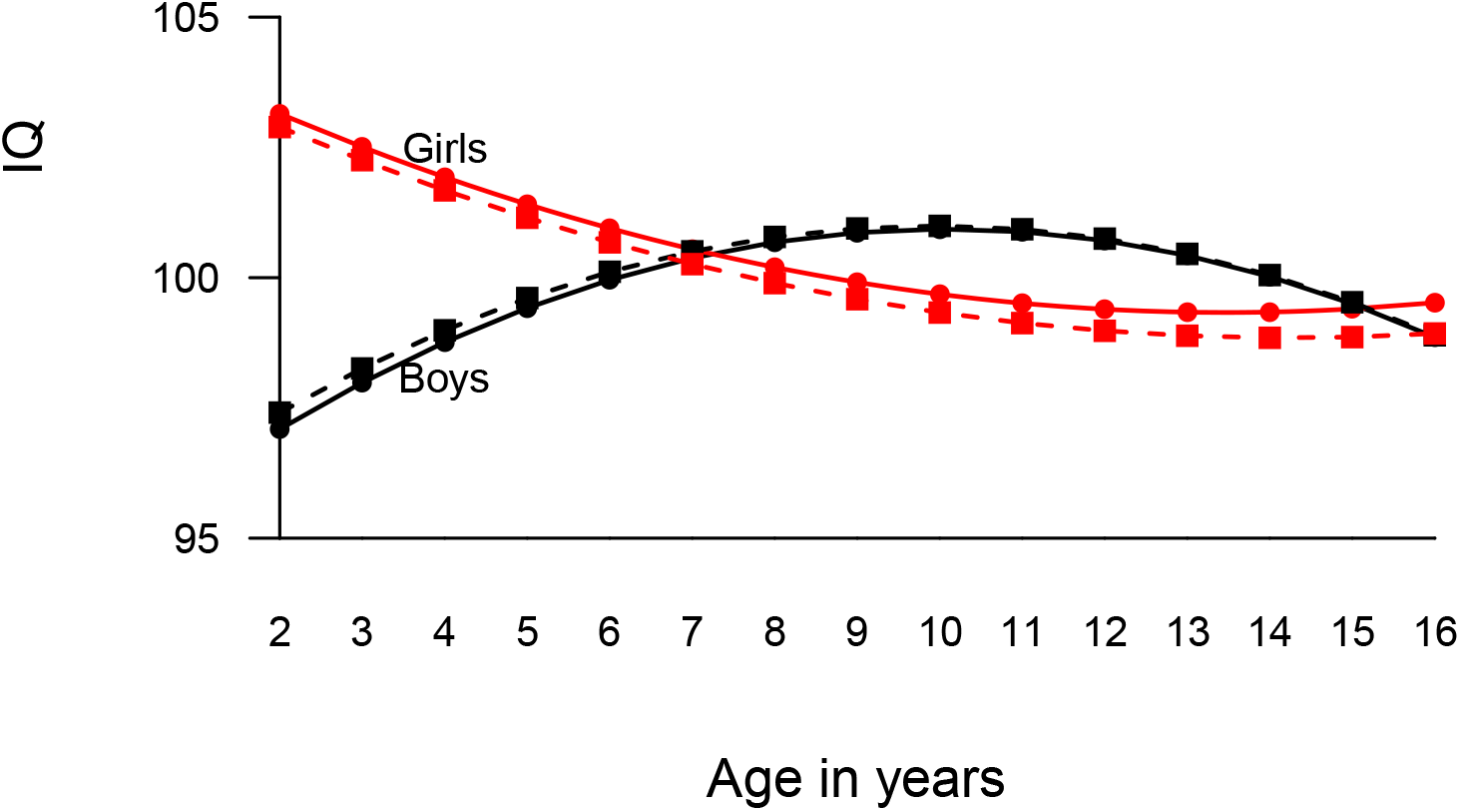
Differences in latent cognitive growth in boys and girls from TEDS from toddlerhood through adolescence. Note: Straight lines represent data from the exploration samples; dotted lines represent data from the replication samples. Boys' trajectories are marked in black; girls' in red.

The difference between the ‘restricted’ and ‘unrestricted’ model across boys and girls showed significant differences in cognitive development in both subsamples of twins (χ²_*difference*_ (12) = 272.05 (twin 1) and 250.94 (twin 2); *p* < .001 for both). Fig 1 illustrates the gender difference in cognitive development. Compared to girls (103.15/ 102.89 with SE .28/ .28 across both subsamples of twins), boys had a lower starting point in IQ (97.09/ 97.41 with SE .30/ 29). However, this disadvantage diminished over time, with boys’ positive slope term (.95/ .90 with SE .09/ .09) and negative quadratic term (-.06/-.06 with SE .01/ .01) resulting in an upward growth curve up to early teenage and a smaller downward trend thereafter. By comparison, a slightly U-shaped curve emerged from girls’ negative slope (-.67/-.66 with SE .08/ .08) and their positive quadratic term (.03/ .03 with SE .01/ .01).

### Breastfeeding and Latent Cognitive Growth

Table 2 shows the regression parameters associated with SES, mothers’ age at birth, gestational age and breastfeeding in boys and girls with their corresponding confidence intervals. Results did not differ across subsamples of twins (model fit differences with *p* > .01 in all cases) and thus results are reported for only one subsample of twins. Regression coefficients of SES and gestational age on the intercept, slope and quadratic term were associated with confidence intervals excluding zero in both boys and girls. By comparison, mothers’ age at birth was only significantly associated with the intercept but not with the LGC factors that represented long-term changes in intelligence. In girls, the confidence interval of the regression parameter between breastfeeding and intercept excluded zero but not in boys. Boys’ and girls’ confidence intervals overlapped greatly for the regression parameter of breastfeeding on the IQ intercept, with the unstandardized point estimate being identical, suggesting that the effect did not differ for boys and girls. Furthermore, confidence intervals for breastfeeding regression parameters with slope and quadratic term included zero. In summary, breastfeeding was not reliably associated with IQ latent growth factors and cognitive growth trajectories after adjusting for relevant covariates.

**Table 2:** Latent growth curve parameters for boys and girls in TEDS.

	B_i_	ß_i_	CI_i_	B_s_	ß_s_	CI_s_	B_q_	ß_q_	CI_q_
		**Girls (N = 3,053)**							
SES	2.53	.14	1.58 to 3.48	0.70	.19	0.43 to 0.97	-0.03	-.14	-0.05 to -0.01
Age	-0.17	-.06	-0.31 to -0.03	0.03	.05	-0.01 to 0.07	< .01	.05	-0.01 to 0.01
Gestational age	0.42	.08	0.18 to 0.65	-0.15	-.13	-0.22 to -0.09	.01	.14	0.01 to 0.01
Breastfeeding	1.83	.07	0.64 to 3.01	0.09	.02	-0.25 to 0.43	< .01	.01	-0.02 to 0.02
		**Boys (N = 2,729)**							
SES	2.55	.14	1.54 to 3.57	0.90	.23	0.59 to 1.21	-0.04	-.20	-0.07 to -0.02
Age	-0.26	-.10	-0.41 to -0.12	0.02	.03	-0.03 to 0.06	<. 01	-.01	-0.01 to 0.01
Gestational age	0.59	.11	0.35 to 0.83	-0.13	-.11	-0.21 to -0.06	.01	.13	0.01 to 0.01
Breastfeeding	1.83	.04	-0.14 to 2.41	0.09	.02	-0.30 to 0.47	< .01	.01	-0.02 to 0.03

*Note*.

i, s, and q refer to intercept, slope and quadratic term, respectively

B refers to unstandardized coefficients, ß refers to the standardized coefficients; and

CI refers to 95% confidence interval of the unstandardized estimate (i.e. B). Coefficients are based on one subsample of randomly selected twins of a pair from TEDS.

## Discussion

The aim of the current investigation was to test if breastfeeding is positively associated with cognitive development from age 2 to 16 years in a prospective cohort study of twins from the United Kingdom. Specifically, we sought to confirm previous findings that suggested breastfeeding’s nutritional benefits for cognitive development occurred early on and persisted over time but that breastfeeding was not associated with long-term cognitive gains [4]. Our results only provide partial support for these previous findings. After adjusting for covariates, girls who had been breastfed scored significantly higher IQ in early life at the age of 2 years compared to girls who had not been breastfed. However, the observed effect was statistically weak and not observed in boys. In line with Huang et al.’s findings, breastfeeding was not associated with slope or quadratic term in either boys or girls in fully adjusted models, suggesting that any associations between breastfeeding and long-term cognitive development are likely to be attributable to the influence of other variables that tend to co-occur with breastfeeding. In principle, our findings suggest no substantial association between breastfeeding and early life intelligence, after adjusting for covariates, echoing the conclusions of a recent review of all relevant studies in this area [1].

We observed significant gender differences in cognitive development that reflected an IQ advantage of girls over boys in early life and differently shaped developmental curves over time. However, gender differences in cognitive development diminished over time and were hardly noticeable after the age of 5 years. Our findings concur with reports about gender differences in cognitive abilities and in brain anatomy [26, 27], but additional longitudinal data are needed to replicate the patterns of gender differences in cognitive development that we observed here.

### Limitations

Our study has many strengths, including the size and nature of its sample and the high frequency of assessments of intelligence over the study period, but it is also not without weaknesses. For example, alternative assessment methods were employed over the course of the study to measure intelligence, including parent-, phone- and web-administered tests, to afford the repeated cognitive assessment of an extremely large sample like TEDS. Although the measures used in TEDS have been previously shown to produce reliable and valid IQ estimates [5, 10, 11], the differences in administration methods are likely to introduce additional measurement error. In separate analyses we tested alternative latent growth curve models that only included IQ assessment data from age 7 to 16 years. The model and factor parameters were similar to those derived from models based on IQ scores from age 2 to 16 years (i.e. including data from parent-administered tests), suggesting that alternative IQ test administration methods do not invalidate our findings. That said, the risk of residual confounding still remains because only a limited number of potentially relevant covariates are available in TEDS, specifically mothers’ age at birth and gestational stage. Thus, it is possible that additional unmeasured confounders, such as parental IQ [3], will affect any association between breastfeeding and cognitive growth trajectories. Furthermore, the risk of residual confounding remains even though our SES measure included parental educational achievement, which has been considered an excellent proxy of IQ [28]. It has been emphasized that a study of twins discordant for breastfeeding would constitute the ideal research design to minimize the effects of residual confounding [1]. However, this is not possible in TEDS, as the discordance of twins for breastfeeding was not recorded. In this context, an additional limitation of the current study is the assessment of breastfeeding, which was based on a single item measure and relied on the information that mothers provided two years after the twins’ birth. While such data are generally thought to be reliable [7], they are subject to measurement error and recall bias.

## Conclusions

In a large sample of British children from TEDS, girls who had been breastfed had a slight advantage in early life cognitive ability after adjusting for covariates; however, the effect was statistically very weak and not significant in boys. Furthermore, breastfeeding was not associated with IQ gains from early life through adolescence for both boys and girls. One might understand these findings to support the notion that breastfeeding has nutritional benefits for intelligence in the first few years of life given that breastfeeding was slightly associated with early life intelligence but not with later cognitive growth. However because the observed effects were weak and at best modest, we interpret the findings as evidence for the lack of any benefits of breastfeeding on cognitive development from early life through adolescence.
